# Practical Anatomy

**Published:** 1912-09

**Authors:** 


### Reviews of Books.

**Practical Anatomy.** By F. G. PARSONS, F.R.C.S., and WILLIAM WRIGHT, M.B., D.Sc., F.R.C.S. In two volumes. Pp. xvi, 467; vii, 382. London: Edward Arnold. 1912.

The authors are to be congratulated upon this modern and truly "practical" manual of Practical Anatomy. It is, perhaps, the first attempt that has been made in this country to write a work of that class upon common-sense lines.

In their Preface the authors state that "they began the book with the idea of leaving out as many facts of anatomy as they dared, because they felt that the modern medical student, with only some fifteen months at his disposal, could not be expected to master the whole of anatomy, or to exercise any sound choice as to which parts of it he should neglect. They justly claim that after deciding how much they could leave out, their endeavour has been to lay stress, firstly on the technical methods of displaying the various structures successfully; and, secondly, on the position and relation of structures which experience shows are of practical importance." The aims thus set forth in the Preface have on the whole been adhered to, and the work is thus a distinct advance on some of the older manuals, in which structures such as the otic ganglion, or the fissures of the cerebellum were extensively treated, and matters of such vast importance as the course of the lymphatics of the tongue and breast, or the surface markings of abdominal viscera, received very scant notice, or were ignored altogether.

The directions for the dissector are explicit, and are a prominent feature of the work. This again stands in happy contrast with many manuals of Practical Anatomy, in which the directions are few, are in small print, and are hidden by pages of verbiage, the proper place for which is in the Text Book. The style is pointed and clear, and many facts are presented in a new and fascinating light. In place of the old-fashioned dissection from above, from below, and from the side, to display the pelvic fascia and its innumerable complications, the authors have incurred a debt of gratitude from every medical student by denying its existence as a definite fascia, except on the inner side of the obturator internus muscle (parietal layer of pelvic fascia), and on the upper surface of the levator ani muscle, close to the os pubis (visceral layer of the pelvic fascia), the remainder being condensed cellular tissue, from which there is no difficulty in producing the orthodox arrangement by manipulation. This view the authors also hold with regard to the so-called deep cervical fascia. Thus they state that it does not form a definite sheath round the carotid artery, jugular vein, and vagus nerve, but that such a sheath is formed by manipulation from the cellular tissue surrounding these structures. A useful addition in the section devoted to the abdomen is the dissection to expose the kidney from behind. The illustrations are mostly diagrammatic, and are all original. For the most part they are not intended to illustrate the stage of the student's dissection, and thus the authors hope that a sense of topography may be acquired. The book is not entirely free from printers' errors ; in one or two places the omission of the necessary comma or semi-colon makes the author's meaning rather unintelligible. In their Preface the authors state that they do not consider the time yet ripe for the exclusive adoption of the revised nomenclature, and that they do not dare to send a student into the wards, believing that the radial nerve is a structure in the upper arm, when to his surgeon it is a structure in the forearm. The objects of the new nomenclature are only too frequently lost sight of. It is most important to realise that the Latin names are for international use only. Professor Krause, the editor of the *Basle Nomenclature*, asserts in stating the objects held in view by the commission, "Each nation using the Latin names can translate them in the way that seems best to it."

To alter the national nomenclature of anatomy is even more impracticable than the reformed spelling of Sir Isaac Pitman. For instance, we cannot expect the student to substitute for internal pterygoid plate of seven syllables—lamina medialis processus pterygoidei, which contains fifteen syllables, and it would be contrary to the objects of the International Commission to do so.

In conclusion, we heartily recommend this book to the notice of the student, and wish it the success it so thoroughly deserves.

